# A Confirmed Case of Xylazine-Induced Skin Ulcers in a Person Who Injects Drugs in Miami, Florida, USA

**DOI:** 10.21203/rs.3.rs-3194876/v1

**Published:** 2023-07-26

**Authors:** Peyton V. Warp, Maia Hauschild, Hansel E. Tookes, Katrina Ciraldo, David P. Serota, Irasema Cruz

**Affiliations:** University of Miami Miller School of Medicine; University of Miami Miller School of Medicine; Division of Infectious Diseases, Department of Medicine, University of Miami Miller School of Medicine; Department of Family Medicine, University of Miami Miller School of Medicine; Division of Infectious Diseases, Department of Medicine, University of Miami Miller School of Medicine; IDEA Exchange, University of Miami Miller School of Medicine

**Keywords:** Xylazine, opioid use disorder, injection drug use, wounds, xylazine test strip, harm reduction

## Abstract

**Background:**

Xylazine is an alpha-2 adrenergic receptor agonist that has emerged as a contaminant in the street drug supply of fentanyl. Xylazine use may be suspected in naloxone-resistant overdoses and atypical, chronic wounds in people who inject drugs (PWID). This case is unique because it is the first case to our knowledge describing wound care for a xylazine-induced wound with a confirmatory xylazine test strip (XTS) in the setting of a syringe services program (SSP) and in the state of Florida.

**Case Presentation:**

A 43-year-old woman with a past medical history of severe opioid use disorder and stimulant use disorder presented to a student-run clinic at the IDEA Miami SSP for wound care. She had multiple ulcerations diffusely over her bilateral forearms with surrounding erythema and warmth. Seven weeks later, she presented to clinic again for wound care because her wounds had progressed. At this visit, a XTS was used to confirm the presence of xylazine in her urine. Wound care management and harm reduction strategies employed at both visits are discussed below. Wound outcomes are unknown as the patient has not returned to clinic.

**Conclusions:**

Many PWID at highest risk for acute and chronic health consequences of xylazine-adulterated fentanyl do not have access to healthcare outside of low barrier clinics and SSPs due to lack of insurance or mistrust of the traditional healthcare system. There is an urgent need for access to XTS for PWID and clinical practice guidelines for the treatment of xylazine-related wounds in outpatient clinics.

## Background

Xylazine is an alpha-2 adrenergic receptor agonist that is a non-opioid drug used for sedation and muscle relaxation in veterinary medicine.^1^ Xylazine is not approved for human use in the United States; however, it has entered the street drug supply as an additive to fentanyl, heroin, and other substances.^2^ Xylazine blocks the release of norepinephrine, resulting in hypotension, bradycardia, respiratory depression, drowsiness, and amnesia. Since xylazine is not an opioid, its effects are not reversed by naloxone, making it much more challenging to treat overdose events. Physicians should have a high clinical suspicion for xylazine-adulterated fentanyl in accurately diagnosing naloxone-resistant overdoses and non-healing, necrotic ulcers in people who inject drugs (PWID). Xylazine first emerged in the early 2000s in Puerto Rico and entered the continental United States a few years later in Philadelphia and Connecticut.^3^ Street-level distributors may use xylazine, also known as “tranq,” as a bulking agent to extend the supply of their other drug products.^4^

In addition to xylazine’s physiologic effects, it is also associated with necrotic skin ulcerations.^5,6^ The mechanism of injury is thought to be directly related to its vasoconstricting effect on local blood vessels, resulting in decreased skin perfusion.^7^ Decreased skin perfusion further leads to impaired healing of wounds and increased risk of superinfection. When coupled with repeated trauma of injections, xylazine can lead to extensive ulceration. In a study conducted in Puerto Rico, it was reported that people who used xylazine had a significantly higher prevalence of skin ulcers (38.5% vs 6.8%) and poorer health than those who did not use xylazine.^8^ Xylazine-induced skin ulcers may appear diffusely throughout the body, even at sites distant from the injection site. These ulcers are typically progressive, large, and necrotic.^9^

Drug-checking has been utilized in medical and community settings to determine the contents of drugs since the 1990s.^10^ This low-barrier harm reduction method has been associated with greater adoption of safer drug use practices.^11^ Unfortunately, xylazine testing is not widely accessible, so PWID are largely unaware of the contents of their drugs. Due to the continuous geographical spread and increasing prevalence of xylazine-adulterated fentanyl, lateral flow immunoassay xylazine test strips (XTS) have become available to allow for rapid, field-based drug testing. XTS manufactured by BTNX, a Canadian biotechnology company, demonstrated high sensitivity (100%), specificity (85%) and precision (91%) in an evaluation conducted by a Philadelphia-based forensic laboratory.^12^

Here we present a representative case of xylazine-induced skin ulcers managed through a student-run free clinic housed in a harm reduction organization.

## Case Presentation

The patient was a 43-year-old woman with severe opioid use disorder (OUD), stimulant use disorder, and a history of infectious complications from injection drug use as well as 3 overdoses. The patient had a 6-year history of injecting fentanyl and cocaine. She was experiencing houselessness in Miami, FL.

**Figure 1:**
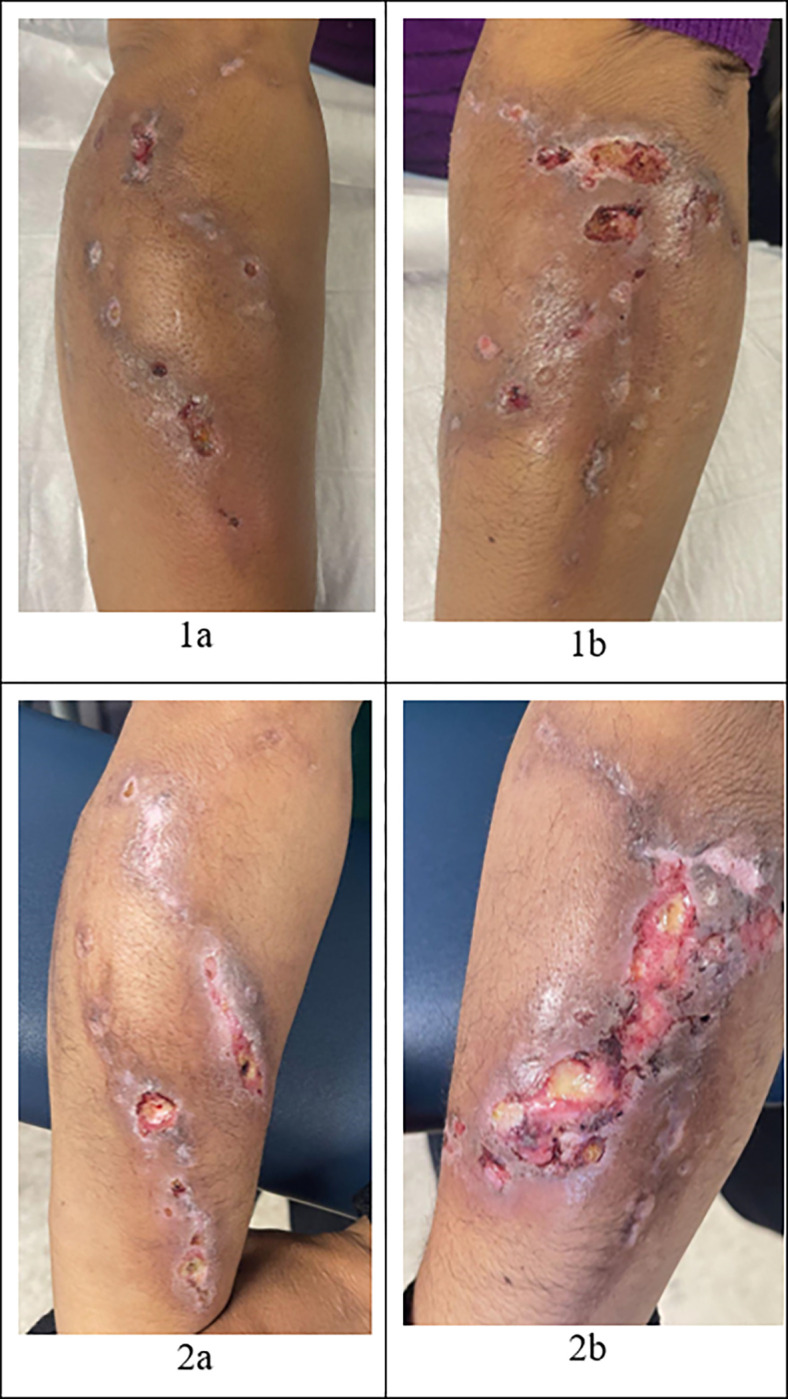
Xylazine-induced wounds

She presented to a free student-run clinic at the IDEA Miami syringe services program (SSP) for wound care. During that time, she was injecting 0.25 g of intravenous fentanyl/cocaine per day in her hands and arms. At this visit, she reported ulcers on bilateral upper extremities. She stated that she had never had wounds like these before despite injecting drugs for 6 years. She described the wounds starting as closed areas of hypopigmentation, then gradually opening, and producing foul-smelling yellow drainage. She noted that when the wounds began, she had a fever that subsided after one day. She denied night sweats, chills, and lymphadenopathy. On a physical exam, she had multiple ulcerations, with the largest being 4 cm in diameter, diffusely over her bilateral forearms with surrounding erythema and warmth (see Fig. 1: 1a, 1b). The wounds were cleaned with saline and dressed with petroleum-impregnated gauze. The patient was instructed to clean the wounds with water and soap daily and replace the petroleum-impregnated gauze dressing. She was also prescribed 100 mg doxycycline oral tablet to be taken twice per day for 14 days.

Seven weeks later, she presented to clinic once again for wound care. She stated she continued injecting drugs and her wounds had progressed (see Fig. 1: 2a, 2b). The wounds were warm, red, and painful and she had a subjective fever. She also noted that new ulcers had formed on her bilateral lower extremities. On physical exam, she had multiple large ulcerations on her bilateral forearms with surrounding hyperpigmentation and 2 small ulcerations with a necrotic center on the lateral aspect of her bilateral knees. The patient denied injecting in her legs. She stated that she had successfully finished her previously prescribed course of antibiotics but was not consistent with cleaning her wound or changing the dressing. At this visit, her urine was tested for xylazine using the Rapid Response^™^ XTS (Liquid / Powder), which is a rapid visual immunoassay for the qualitative, presumptive detection of xylazine in liquids or powders at the cut-off concentration of 1000 ng/mL. This test strip was positive, indicating the presence of xylazine. The wounds were cleaned with saline solution and dressed the same as last time, and she was given the same wound care instructions. She was also prescribed 80/400mg trimethoprim/sulfamethoxazole oral tablet to be taken twice per day for 14 days.

## Discussion and Conclusions

The IDEA SSP obtained XTS and began testing patients who had tested positive for fentanyl for xylazine on April 27, 2023 and as of July 22, 2023, 55.8% (24/43) of tests have been positive. These tests confirm the presence of xylazine-adulterated fentanyl in Miami. While there have been reports of intentional xylazine use,^13^ with patients reporting that xylazine “extends the high’’ and “gives the [fentanyl] more of a heroin effect,” recent studies emphasize PWID’s overwhelming concern about the adulterated fentanyl supply.^14^ Xylazine’s sedating effects leave unhoused PWID vulnerable to robbery and assault, and one study stated that xylazine-induced wounds are a primary concern among PWID.^3^ The rising prevalence of xylazine-adulterated fentanyl is a concerning trend that may be changing the risk profile of substance use, altering substance use patterns, and affecting the treatment response of individuals with suspected overdose events.^15^

To prevent unwanted exposure to xylazine and the novel health risks associated with a xylazine-adulterated fentanyl supply, harm reduction and healthcare efforts must focus on making XTS available to PWID. Evidence suggests a favorable attitude of PWID toward XTS; in a recent study, 100% of PWID interviewed about xylazine in Philadelphia were interested in hypothetical XTS.^14^ The acceptability and utility of fentanyl test strips supports widespread dissemination of XTS to PWID, as fentanyl test strips have been reported to change drug use behavior and improve perceived overdose safety.^16^ Harm reduction programs, such as the IDEA SSP, play an important role in linking PWID to healthcare services. Low barrier access to lifesaving medications for OUD (e.g. buprenorphine) could also mitigate harm associated with xylazine-adulterated fentanyl by supporting recovery of OUD.^17^ Reducing barriers to accessing XTS, medications for OUD, and SSPs is a necessary step in mitigating harm associated with a xylazine-adulterated fentanyl supply.

Regarding xylazine-induced wounds, there is an emergent need for clinical practice guidelines to aid treatment plans.^2^ In this case, we approached treating the wounds with petroleum-impregnated gauze to keep the area moist. Petroleum-impregnated gauze is a semi-open dressing consisting of a primary layer facing the wound, a secondary layer containing the petroleum-infused absorbent gauze and padding, and a third layer of adhesive. With this design, fluid is able to seep through the first layer and be collected in the second layer, allowing for hydration of the wound bed and surrounding tissue. Studies have shown that wounds kept in a moist environment heal more rapidly compared to wounds kept dry.^18,19^ However, given the many social barriers this patient faced, it was difficult to achieve optimal wound cleaning and dressing. Additionally, more frequent visits could have allowed adjustments to the treatment plan to best suit her needs and achieve optimal outcomes. Another limitation in the treatment of this patient was the limited resources available at this student-run free clinic.

In conclusion, given the rising prevalence of xylazine-adulterated fentanyl, there is an urgent need for access to XTS for PWID and clinical practice guidelines for the treatment of xylazine-induced skin wounds for healthcare professionals. Next steps include epidemiologic research studying the geographic spread of xylazine-adulterated fentanyl, its clinical effects, and implementation of overdose prevention strategies (e.g. XTS) delivered in settings frequented and trusted by PWID such as harm reduction programs.

## Data Availability

Not applicable.

## Abbreviations

PWID: people who inject drugs
XTS: xylazine test strips
OUD: opioid use disorder
SSP: syringe services program
